# The decision-making process and experiences of women returning to work after parental leave: a qualitative systematic review protocol

**DOI:** 10.1186/s13643-025-02778-3

**Published:** 2025-02-07

**Authors:** Safiye Sahin, Sabine S. Dunbar, Gurmeet Sehgal, Lisa R. Roberts, Jan M. Nick

**Affiliations:** 1 School of Nursing, Loma Linda University, Loma Linda, CA USA; 2 Loma Linda University Library, Loma Linda, CA USA; 3 LLUH Center for Evidence Synthesis, Loma Linda University Health, Loma Linda, CA USA

**Keywords:** Maternity leave, Gender roles, Career re-entry, Work-family policies

## Abstract

**Objective:**

To investigate the decision-making process of women returning to work after maternity leave or parental leave and explore the influence of cultural norms and societal expectations on their choices. Additionally, we seek to understand the lived experiences of the women in this context.

**Introduction:**

Cultural norms and societal expectations significantly affect women’s decisions regarding post-childbirth employment. However, a comprehensive understanding of these influences on women’s experiences returning to work after parental leave is lacking.

**Inclusion criteria:**

We will include qualitative studies examining women’s decision-making processes and experiences of returning to work after parental leave, with a focus on the influence of cultural norms and societal expectations. Studies from diverse cultural and geographical settings, including peer-reviewed journals and gray literature, will be considered without restrictions on publication date or language.

**Methods:**

The review will adhere to the JBI approach for qualitative systematic reviews. Major academic databases and search engines, such as CINAHL, PubMed, and Google Scholar, will be used. Study selection will involve screening titles and abstracts for relevance, followed by a full-text assessment against inclusion criteria by two independent reviewers. Critical appraisal using the JBI Critical Appraisal Checklist for Qualitative Research will evaluate study rigor. Data extraction will be conducted by two independent reviewers, using the standardized JBI data extraction tool within JBI SUMARI, to identify key themes and findings related to the women’s decision-making process and lived experiences of returning to work after parental leave. The meta-aggregation approach will be utilized to synthesize findings, with confidence assessed through study quality and consistency. Any methodological deviations will be documented. Findings will be graded using the ConQual approach and presented in a summary of findings table.

**Discussion:**

By synthesizing data from different cultural contexts, this review will help bridge the gap in understanding how these factors influence women’s choices. Rigor in the review will be ensured through the process of study selection, appraisal, and synthesis using the JBI approach. The findings will provide challenges faced by women and inform policies to help support their transition back to work.

**Systematic review registration:**

PROSPERO CRD42024546633.

## Introduction

The average labor force participation rate of women aged 15 and above, is estimated to be about 47.6% [1]. According to the International Labor Organization, almost all countries continue to witness a lower participation rate of women in the workforce compared to men, with the gender gap higher than 50 percentage points in some regions [2]. The lower participation rate of women in the workforce compared to men can be attributed to a variety of factors. According to a survey from the European Institute for Gender Equality, in 2023, women with at least one child under 6 had an employment rate 10.5 percentage points lower than women without children, while men with children under 6 had an employment rate 8.2 percentage points higher [3]. There are no recent official data on mothers returning to work after maternity leave worldwide; however, studies suggest the rate varies based on factors such as country, company policies, industry, and individual circumstances [4, 5]. For example, Choi found that 64.8% of mothers with children younger than 1 year returned to work within 12 months of leave in 2009 while it was 59.6% in 2019 in Canada [4].

Cultural norms and social expectations often assign caregiving and family duties to women. This limits their ability to work full-time or advance in their careers [6]. Additionally, insufficient family-friendly policies, such as paid parental leave and affordable childcare, exacerbate these challenges [5]. These challenges require comprehensive support from employers, policymakers, and society to ensure that women have the resources and opportunities to balance their work and family responsibilities without sacrificing their careers.

Governments worldwide are taking proactive steps to address the lower rates of female workforce participation by implementing policies for supporting women. Of these programs, maternity leave is one of the key mechanisms for women in the transition to motherhood [7]. Maternity leave is a job-protected absence around the time of childbirth or adoption [8]. The ILO recommends at least 14 weeks of leave period, with the option to combine pre- and post-birth leave in many countries [9]. Nearly all Organization for Economic Co-operation and Development (OECD) countries offer financial support associated with maternity leave [8].

Similarly, parental leave, designed for employed parents, often follows maternity or paternity leave, extending support for childcare [10]. Maternity and parental leave policies vary globally, with some countries integrating maternity leave within parental leave regulations, like Australia, Iceland, New Zealand, Norway, and Sweden. In certain nations, specific periods of parental leave are gender-specific, while others, like Austria and Germany, incentivize joint parental leave usage with additional paid weeks [8, 11]. In our study, we focus on women-specific leave policies, including both maternity and parental leave.

Gendered social norms and traditional gender roles have a powerful influence on women’s labor force participation and shape their experiences at work [12]. From an early age, individuals are socialized into specific gender roles, which define appropriate behaviors and expectations of what constitutes masculinity and femininity [13]. Women tend to be tasked with primary responsibility for caregiving and household duties, whereas men are expected to be the principal breadwinners [14]. These deeply ingrained societal norms not only perpetuate gender disparities in caregiving responsibilities but also contribute to obstacles to women’s full economic participation [15]. Women may face discrimination, bias, and limited career opportunities as they move through the labor market shaped by traditional gender roles [16]. Furthermore, these expectations of putting family first over careers can affect their decision-making process.

Returning to work after parental leave is a crucial moment for many women. It involves a range of emotions, challenges, and goals that shape their experiences [5]. In navigating this transition, they engage in personal motivations, financial considerations, and societal expectations [17]. For some, going back to work brings a sense of empowerment and fulfillment, allowing them to pursue their professional goals while maintaining a sense of identity beyond motherhood. However, for others, returning to work may evoke feelings of anxiety, guilt, and uncertainty as they navigate how to balance career ambitions with caregiving responsibilities [18]. Moreover, the work environment can pose a myriad of challenges, from negotiating work time arrangements to combating gender bias and discrimination [6]. Understanding women’s decision-making process and lived experiences of returning to work after parental leave is crucial in informing policies and practices that promote equality and work-life balance.

Although existing primary studies explore the decision-making process and lived experiences of women as they re-enter the workforce following parental leave [5, 17], there is still a lack of a comprehensive synthesis that captures the breadth and depth of women’s experiences across diverse settings and cultural contexts. These primary studies, while providing valuable insights, often focus on different aspects of women’s experiences in varying geographical and cultural contexts, making it difficult to integrate and compare findings. Additionally, the studies use diverse methodologies, such as phenomenology, grounded theory, and ethnography, which further complicates the synthesis of results. Many studies examine specific facets of women’s experiences, such as work-life balance or career aspirations, but rarely offer a holistic view that encompasses all the challenges, motivations, and aspirations shaping women’s decisions to return to work after parental leave. As a result, there is a critical need for a qualitative meta-synthesis. Such a synthesis, which integrates the findings from these primary studies, will provide a holistic view that informs policies, practices, and interventions aimed at supporting women in the workforce and promoting gender equality.

A preliminary search across various databases including PROSPERO, MEDLINE, the Cochrane Database of Systematic Reviews, and the JBI Evidence Synthesis yielded no current or ongoing systematic reviews specifically addressing the qualitative decision-making process and lived experiences of women returning to work after maternity/parental leave. While there is an existing systematic literature review on the lived experiences of women returning to work after maternity leave [19], it includes both quantitative and qualitative primary studies and describes the existing literature. By contrast, our qualitative systematic review will aim to conduct a meta-synthesis analysis of women’s lived experiences and decision-making when returning to work following parental leave. Our review will include only qualitative and mixed-methods primary studies and follow the Joanna Briggs Institute (JBI) methodology.

The objective of the review is to explore how women navigate the decision-making process regarding return to work after parental leave, with a particular focus on the influence of cultural norms and societal expectations surrounding motherhood. In addition to this, it aims to capture the lived experiences of women as they return to work after parental leave.

### Review question(s)

1. How do women navigate the decision-making process regarding whether to return to work after parental leave?
  - How do cultural norms and societal expectations surrounding motherhood influence women’s decisions about returning to work after parental leave?
2. What are the lived experiences of women returning to work after parental leave?

### Inclusion criteria

#### Participants

Participants included in this review will be women who have taken paid or unpaid parental leave (any duration) and subsequently returned to work in a part-time job (regardless of hours worked) or a full-time job, and remote work, or in-person work. Additionally, adoption will be included. There will be no restrictions based on relationship type (e.g., heterosexual or same-sex relationships), age, ethnicity, multiple-gestation, or socioeconomic status.

Exclusion criteria will involve studies focusing solely on fathers or individuals who have not taken parental leave or suffered a stillbirth or neonatal death, as well as studies where participants’ experiences of returning to work after parental leave are not explicitly explored or discussed.

#### Phenomena of interest

This review will consider studies that explore and provide detailed descriptions of the women’s decision-making process, cultural norms, societal expectations, and lived experiences returning to work after parental leave.

#### Context

The context for this review encompasses diverse cultural and geographic settings, recognizing that women’s decision-making process, cultural norms and societal expectations, and experiences of returning to work after parental leave may vary based on societal norms, cultures, and legal frameworks. Studies conducted in various countries and regions will be considered to capture these contextual nuances. Additionally, the review will acknowledge specific sub-cultural factors, such as racial and gender-based identities, as they may influence women’s decision-making processes and experiences in the workplace. The context will also include home settings for remote workers. Exclusion criteria will involve studies that include incarcerated mothers who may not have a choice regarding work.

#### Types of studies

This review will consider qualitative studies, as well as mixed methods studies that draw on the decision-making process and experiences of women returning to work after parental leave, including, but not limited to, designs such as phenomenology, grounded theory, ethnography, and focus groups.

## Methods

The proposed systematic review will be conducted in accordance with the JBI methodology for systematic reviews of qualitative evidence [20]. We will follow the Preferred Reporting Items for Systematic Reviews and Meta-Analysis Protocols (PRISMA-P) guidelines to ensure thoroughness in protocol standards [21]. Furthermore, this review protocol has been submitted for registration in PROSPERO, with the registration status currently pending.

### Search strategy

The search strategy will aim to locate both published and unpublished studies. A three-step search strategy will be utilized in this review. First, an initial limited search of MEDLINE (PubMed) was undertaken to identify appropriate subject headings and analyze text words in the titles and abstracts of relevant articles. The text words contained in the titles and abstracts of relevant articles, and the index terms will be used to describe the articles, and along with controlled vocabulary, a full search strategy will be developed for the full review. The search strategy, including all identified keywords and index terms, will be adapted for each included database and/or information source. A librarian assisted with developing the initial search strategies and will assist with the final search strategy and managing search results (see Appendix 1 for a sample search strategy for MEDLINE via PubMed).

During the second step, after finalizing all identified keywords and index terms, a simultaneous search will be undertaken to increase the sensitivity of the results. Databases and associated platforms to be searched will include the Cochrane Database of Systematic Reviews (Cochrane Library), Embase (Elsevier), MEDLINE (PubMed), CINAHL (EbscoHost), PsycInfo (EbscoHost), SocIndex (EbscoHost), Web of Science Core Collection, and JBI Evidence Synthesis. For gray literature, the team will search DANS EASY for OpenGrey archived literature, and Google Scholar for peer-reviewed and non-peer-reviewed publications using private “incognito” settings in Google Chrome to minimize bias.

In the third step of the search strategy, the reference lists of all included sources of evidence will be screened for additional studies. Only qualitative research (e.g., phenomenology, grounded theory, and ethnography studies) will be included in this review. Studies published in any language and from all publication years will be included.

### Study selection

After completing the search, all identified citations will be gathered and organized using Endnote 20 (Clarivate Analytics, PA, USA), with duplicates removed. Potentially relevant studies will then be retrieved, and their citation details imported into the JBI System for the Unified Management, Assessment and Review of Information (JBI SUMARI; JBI, Adelaide, Australia) [22].

The screening and selection process will follow a three-step approach to ensure accuracy and thoroughness. Initially, all screeners will be involved in a pilot test, and independently screen 25 titles/abstracts using inclusion/exclusion criteria. Upon reaching a 90% agreement between all raters, any two team members will proceed to assess titles and abstracts independently.

The second step involves reviewing the full text of selected articles. Studies not in English will be translated using the DeepL translation software program (DeepL, Cologne, Germany) before full-text screening. Two independent reviewers will assess the full text against the inclusion criteria, and document reasons for exclusion which will be recorded and reported in an Appendix.

The third step involves assessing methodological quality using JBI critical appraisal tools, with two independent reviewers determining final study inclusion. Any disagreements between reviewers will be resolved through discussion or by involving additional team members. The results of the search and study inclusion process will be fully reported in the systematic review and depicted in a Preferred Reporting Items for Systematic Reviews and Meta-analyses (PRISMA 2020) flow diagram [23].

### Assessment of methodological quality

Eligible studies will be critically appraised by two independent reviewers for methodological quality using the standard JBI Critical Appraisal Checklist for Qualitative Research [24]. This tool includes 10 items to determine whether there are statements regarding the alignment of purpose, methodology, and dependability in qualitative research. It provides “yes,” “no”, “unclear” options, and “not applicable.” The “unclear” option will be selected when there is a simplified explanation or text provided in the study, but the specific details are not known.

In addition to methodological quality, this checklist will be also employed for the assessment of methodological quality with respect to the risk of bias for individual studies, relating to selective reporting, publication bias, or bias that could potentially influence the researcher. The assessment will ensure that any biases are identified and resolved in a systematic manner, which will contribute to a more accurate synthesis of the evidence.

Authors of papers will be contacted to request missing or additional data for clarification, where required. Any disagreements that arise between the reviewers will be resolved through discussion, or with a third reviewer. The results of critical appraisal and risk of bias assessment will be reported in narrative form and in a table. The risk of bias table will provide a clear and structured summary assessment of each study, detailing the judgments for key domains and offering an overall risk of bias rating for each study.

Following critical appraisal, studies will be excluded based on predetermined decision rules regarding their methodological quality and risk of bias. Scores will be categorized as follows: 0–4 “yes” responses (0–40%) indicate a high risk of bias; 5–6 “yes” responses (50–60%) indicate moderate-high risk of bias; and 7–10 “yes” responses (70–100%) indicate low risk of bias. Moderate and low-risk bias studies will be included in the review. By only including studies with moderate and low risk of bias, the review aims to produce more credible, high-quality evidence, which will enhance the robustness of its conclusions.

### Data extraction

Data will be extracted from included studies by two independent reviewers using the standardized JBI data extraction tool in JBI SUMARI (Appendix 2). The extracted data will encompass specific details about the study, the populations (e.g., demographic characteristics of participants, length of parental leave, age of child, spouse’s working status), context (e.g., organizational culture, societal norms), culture, geographical location, study methods (e.g., data collection techniques, analysis approach), and the phenomena of interest relevant to the review objective. These may include exploring how cultural norms and societal expectations influence women’s decisions regarding returning to work after parental leave, as well as capturing the lived experiences of women during this transition period.

Findings, along with illustrations, will be extracted verbatim and assigned a level of credibility based on the original study’s interpretation. To minimize errors during data extraction, all reviewers will pilot-test the data extraction tool on three studies. Data will be independently extracted from the included studies by two reviewers to reduce bias.

During the data extraction process, any disagreements between reviewers will be resolved through discussion, and if necessary, with a third reviewer. Authors of papers will be contacted to request missing or additional data, where required. Assumptions about missing or unclear information will be made cautiously, considering the potential impact on the overall findings and interpretations. In addition, any methodological deviations encountered during the review process, such as modifications to inclusion criteria or adjustments in data extraction, will be documented and justified.

### Data synthesis

Qualitative research findings will, where possible, be pooled using JBI SUMARI with the meta-aggregation approach [25]. This will involve the aggregation or synthesis of findings to generate a set of statements that represent that aggregation, through assembling the findings and categorizing them on the basis of similarity in meaning. These categories will then be subjected to a synthesis in order to produce a single comprehensive set of synthesized findings that can be used as a basis for evidence-based practice. Where textual pooling is not possible, the findings will be presented in narrative form. Only unequivocal and credible findings will be included in the synthesis. Mothers’ experiences of decision-making to return to work as well as cultural influences will be synthesized together. The final findings of the decision-making process will be expressed in narrative form.

### Assessing confidence in the findings

The final synthesized findings will be graded according to the ConQual approach for establishing confidence in the output of qualitative research synthesis and presented in a summary of findings table [26]. This summary of findings will encompass the major elements of the review, including the title, population, phenomena of interest, and context specific to the review. Each synthesized finding from the review will then be presented, including the methodological approach, score for dependability and credibility, and the overall ConQual score.

## Discussion

This systematic review protocol aims to synthesize the data from qualitative single studies on the decision-making processes and living experiences of women returning to work following parental leave, focusing on cultural norms and societal expectations. The initial findings of this study indicate that the post-child-delivery employment status of women is crucial in terms of women’s participation in workforce labor. While the literature recognizes the influence of cultural and societal norms on the decision-making process and lived experiences of women returning to work after childbirth, a comprehensive synthesis of qualitative data across different settings, cultures, and contexts is lacking. This review will fill this gap by including qualitative studies from different geographical and cultural backgrounds that could give a wider perspective into how the decision-making processes and experiences of women are shaped.

The strength of this review is that in following the JBI methodology, the selection of studies, critical appraisal, and synthesis will be rigorous. Application of the meta-aggregation approach will make identification of key themes possible while maintaining integrity at a study level. It will ensure the comprehensiveness of the review by including both peer-reviewed literature and gray literature without restriction of language or date of publication.

In addition to its strengths, there will be a risk that the differences in cultural backgrounds or settings might create variation (heterogeneity) in the results or interpretations. In this case, the ConQual approach will serve as a methodological process through which the confidence in the findings will be graded such that any inconsistencies will be carefully analyzed and evaluated.

Overall, this review will contribute to the understanding of decision-making processes and lived experiences of women’s return to work from parental leave. Results that better reflect the needs of women in the process of returning to work in various cultural and social contexts can be used for policy development.

## Data Availability

Not applicable.

## Appendix 1

### Search strategy

Database (platform): MEDLINE via Pubmed

Search conducted on 01/09/2025

**Table:**

Search	Search strategy	Results retrieved
#1	(Return to Work [Mesh] OR “return to work” [tiab] OR “back to work” [tiab] OR employment [Mesh] OR employment[tiab] OR workplace [Mesh] OR workplace[tiab] workforce [tiab] OR rejoin[tiab] OR reintegrate[tiab] OR "Work-Life Balance"[Mesh] OR “Work Life Balance” [tiab] OR "Teleworking"[Mesh] OR “Remote Work*” [tiab] OR “Hybrid Work” [tiab])	13,568
#2	(“maternity leave” [tiab] OR parental leave [Mesh] OR postpartum period [Mesh] OR postpartum[tiab] OR Mothers [Mesh] OR mother*[tiab] OR family leave[tiab] OR “maternal leave” [tiab] OR childbirth[tiab] OR "Parturition"[Mesh] OR Family Leave [Mesh] OR “family leave” [tiab] OR "Adoption"[Mesh] OR adoption[tiab])	522,848
#3	(Decision making [Mesh] OR “decision making” [tiab] OR decision[tiab] OR factor*[tiab] OR enabler[tiab] OR barrier*[tiab] OR family support [Mesh] OR support[tiab] OR facilitator[tiab] OR challenges[tiab] OR Mothers, Psychology [Mesh] OR Women Working, Psychology [Mesh] OR Culture [Mesh] OR culture[tiab] OR social norms [Mesh] OR social conformity [Mesh] OR social[tiab] OR bias[tiab] OR Working conditions [Mesh] OR” working conditions” [tiab] OR “childcare policies” [tiab])	8,108,000
#4	(Qualitative Research [Mesh] OR qualitative[tiab] OR experience*[tiab] OR grounded theory [Mesh] OR ethnography[tiab] OR ethnographies[tiab] OR ethnology [Mesh] OR phenomenolog*[tiab] OR Focus Groups [Mesh] OR “focus group*” [tiab] OR “mixed methods” [tiab])	1,839,603
#5	#1 AND #2 AND #3 AND #4	177

## Appendix 2

### Data extraction instrument

JBI. (Updated in 2024). JBI Data Extraction Form for Qualitative Research. Retrieved from: https://jbi-global-wiki.refined.site/space/MANUAL/4687826/Appendix+2.3%3A+JBI+Qualitative+data+extraction+tool
